# Difference between ejection times measured at two different peripheral locations as a novel marker of vascular stiffness

**DOI:** 10.1371/journal.pone.0187781

**Published:** 2017-11-29

**Authors:** Yurie Obata, Pavel Ruzankin, Dan E. Berkowitz, Jochen Steppan, Viachaslau Barodka

**Affiliations:** 1 Division of Cardiac Anesthesia, Department of Anesthesiology and Critical Care Medicine, The Johns Hopkins University School of Medicine, Baltimore, Maryland, United States of America; 2 Sobolev Institute of Mathematics, Novosibirsk, Russia; 3 Novosibirsk State University, Novosibirsk, Russia; University of Colorado Denver School of Medicine, UNITED STATES

## Abstract

Pulse wave velocity (PWV) has been recommended as an arterial damage assessment tool and a surrogate of arterial stiffness. However, the current technology does not allow to measure PWV both continuously and in real-time. We reported previously that peripherally measured ejection time (ET) overestimates ET measured centrally. This difference in ET is associated with the inherent vascular properties of the vessel. In the current study we examined ETs derived from plethysmography simultaneously at different peripheral locations and examined the influence of the underlying arterial properties on ET prolongation by changing the subject’s position. We calculated the ET difference between two peripheral locations (ΔET) and its corresponding PWV for the same heartbeat. The ΔET increased with a corresponding decrease in PWV. The difference between ΔET in the supine and standing (which we call ET index) was higher in young subjects with low mean arterial pressure and low PWV. These results suggest that the difference in ET between two peripheral locations in the supine vs standing positions represents the underlying vascular properties. We propose ΔET in the supine position as a potential novel real-time continuous and non-invasive parameter of vascular properties, and the ET index as a potential non-invasive parameter of vascular reactivity.

## Introduction

For the clinician it is important to distinguish patients with a compliant vasculature from those with stiff vessels [1]. The gold standard to measure vascular stiffness is carotid–femoral PWV (cf-PWV) [2], which has been recommended by the European Society of Hypertension and the European Society of Cardiology (ESH/ESC) as a marker for arterial damage and by the American Heart Association (AHA)’s Scientific Statement as a surrogate for arterial stiffness [3,4]. However PWV depends not only on intrinsic vascular stiffness but also on vessel wall tension, which dynamically changes with fluctuations in blood pressure (BP) [5,6]. Several attempts have been made to develop a BP independent index of vascular stiffness, such as the Cardio-Ankle Vascular Index (CAVI) and the Arterial Stiffness Index (ASI) [7,8]. However, they are not consistently BP independent in all clinical scenarios [8,9]. Moreover the current technology for PWV measurement does not allow to determine these indices continuously and in real time. Hence there is a clinical need to develop a real time continuous and noninvasive marker of vascular stiffness.

The arterial blood pressure waveform changes as the pulse wave travels across the arterial tree to different peripheral locations [10]. Our group has recently reported that the peripherally measured ejection time, derived from the radial artery blood pressure waveform, consistently overestimates a centrally measured ejection time at lower BPs, slow HRs, and low PWVs [11]. This prolongation represents a direct modulating effect of the vasculature on the pulse waveform. Based on these results and the underlying assumption that the effect of the vasculature on the pulse waveform is the main factor for the changes seen in the peripherally measured ET we hypothesized that ET prolongation will be different at different peripheral locations. Furthermore for subjects with a lower vascular tone (younger subjects with lower blood pressure and PWV), a higher degree of distortion and hence a longer ET prolongation would be observed at more distal measurement sites. The aims of the present study were therefore: (1) to compare ETs obtained peripherally by plethysmograph at different locations for the same heartbeat, (2) to examine the influence of the arterial properties on ET prolongation, by changing the subject position, and (3) to develop a simple and non-invasive method for evaluating vascular properties based on measuring ET prolongation.

## Materials and methods

### Subjects

This study utilized the data derived from the subjects enrolled in the previous study which investigated the effects of posture on pulse arrival to the peripheral vascular beds [12]. The protocol was approved by The Johns Hopkins Medicine Institutional Review Boards (IRB00074229). Inclusion criteria were: Healthy adults, age 21–50 years, both genders. Exclusion criteria were: Subject refusal to participate, known cardiovascular disease of any kind (including hypertension), and pregnancy.

### Study protocol

We measured each subject’s height, weight, and the distance from the sternal notch to 1) the ear lobe, 2) the index finger, with the arm abducted at 90 degrees, 3) the big toe. A standard 3 lead ECG (Bio Amp FE132, ADInstruments, Australia) was placed for continuous monitoring of electrical cardiac activity. Plethysmography sensors (MLT1020EC IR Plethysmograph (ear) and MLT1020PPG IR Plethysmograph (finger and toe), Ad Instrument, Australia) were placed on the left ear lobe, left index finger, and left big toe. We simultaneously recorded lead II of the ECG along with the plethysmograph signals for 30 seconds, in each the standing, sitting and supine position. The ECG and plethysmograph sensors were then removed from the subjects and a blood pressure cuff applied on the right upper arm to record systolic, diastolic, and mean blood pressure (SBP, DBP, MAP) in the standing, sitting and supine position respectively. The ECG and plethysmograph signals were simultaneously converted digitally at 1 kHz (PowerLab4/26, ADInsruments, Australia) and recorded in the LabChart software (LabChart 8, Ad Instruments, Australia).

### Data extraction

We extracted the ejection time (ET) from the difference between the dicrotic notch arrival time (DAT) and the corresponding pulse arrival time (PAT) for each location (ear, finger, and toe) and position (standing, sitting, and supine). The dicrotic notch arrival time (DAT) at each location was obtained by calculating the time delay between the peak of the R wave on the ECG and the dicrotic notch on the plethysmograph waveform. The pulse arrival time (PAT) at each location was obtained by calculating the time delay between the peak of the R wave on the ECG waveform and the initiation of the upstroke on the plethysmograph waveform.

Data extraction was performed by a computer-automated technique as described previously [13–15]. Briefly, we applied standard 60Hz notch and high-pass filters followed by a smoothing procedure. We then extracted the peak of the R wave based on the published algorithm as proposed by Pan and Tompkins [13]. The initiation of the upstroke of the plethysmograph waveform and the dicrotic notch on the plethysmograph waveform were extracted based on the second derivative [14,15]. Periods consisting of over 10 consecutive heartbeats from each position were used for data extraction. Only pulse waveforms with a distinct dicrotic notch, as defined by the automated computer algorithm, were accounted for in the analysis. Mean variables for each location and in each position were calculated using 17±10 waveforms for each subject.

We calculated the time difference between the ET at the toe and the ET at the ear (ΔET_Toe-Ear_), between the ET at the toe and the ET at the finger (ΔET_Toe-Finger_), and between the ET at the finger and the ET at the ear (ΔET_Finger-Ear_) in each position for each subject and the same heartbeat. ΔPAT and ΔDAT were calculated in the same way (ΔPAT_Toe-Ear_, ΔPAT_Toe-Finger_, ΔPAT_Finger-Ear_, ΔDAT_Toe-Ear_, ΔDAT_Toe-Finger_, and ΔDAT_Finger-Ear_). Systolic PWV (PWV_ΔPAT_) was calculated as suggested by Alivon et al. as the vascular path length divided by ΔPAT for each pair of locations and in each position [16]. The vascular path length was calculated as the difference between the two vascular path lengths. For instance, PWV_ΔPAT Toe-Ear_ was calculated as follow, (distance from the sternal notch to the toe—distance from the sternal notch to the ear) / (ΔPAT_Toe-Ear_). We used the same approach to calculate diastolic PWV (PWV_ΔDAT_) based on ΔDAT instead of ΔPAT for each pair of locations and in each position. PWV_ΔDAT Toe-Ear_ was calculated as follow, (distance from the sternal notch to the toe—distance from the sternal notch to the ear) / (ΔDAT_Toe-Ear_). PWV_ΔPAT Toe-Finger_, PWV_ΔPAT Finger-Ear_, PWV_ΔDAT Toe-Finger_ and PWV_ΔDAT Finger-Ear_ were calculated in the same way. We defined “ET index” as the difference between the mean of ΔET_Toe-Finger_ in the standing and supine positions in each individual subject by subtracting the former from the latter. We defined “ΔPWV_Standing-Supine_” as the difference between the mean of PWV_ΔPAT Toe-Finger_ in the standing and supine positions in each individual subject by subtracting the latter from the former. Since PWV_ΔPAT Toe-Finger_ has been validated previously as a good surrogate for arterial stiffness [16], we chose the same locations (toe and finger) to calculate ET index and ΔPWV_Standing-Supine_. We compared ΔET_Toe-Finger_ to PWV_ΔPAT Toe-Finger_ and ET index to ΔPWV_Standing-Supine_ in this study. We calculated ET index as a novel index reflecting the effect of both measurement sites and positions on ET prolongation.

### Statistical analyses

All Data was analyzed using GraphPad Prism version 7 (GraphPad Software, San Diego, California, USA). Based on the results of the previous study, we estimated that a sample of 4 subjects would provide adequate power (80% power for a mean (SD) difference of 53ms (22 ms)) for the change in ET between two locations at an alpha level of 0.05 in a two-sided paired t-test [11]. We report continuous variables as mean ± standard deviation (SD) and categorical variables as proportions. The non-parametric Friedman test and a post hoc Dunn’s multiple comparison test were performed to compare three continuous variables measured in different positions or at different locations. Moreover, we performed a Bland-Altman analysis to assess the difference between ETs at two peripheral locations. The relationship between two continuous variables was assessed using a linear regression analysis. A nonlinear regression analysis was performed to assess the relationships between ΔET and PWV. We classified the subjects into two subgroups according to their percentile rank of the ET index, group 1: above the 50th percentile (n = 5), group 2: below the 50th percentile (n = 6). A Mann-Whitney test was used to compare the continuous variables in group 1 with those in group 2. Fisher’s exact test was used to compare the categorical variables in group 1 with those in group 2. Statistical significance was set at P < 0.05 and all test were two sided. We created a generalized linear model (GLM) to assess if the ΔET and ET index could be explained by age, gender, body mass index (BMI), mean arterial pressure (MAP), heart rate (HR), and PWV.

## Results

We enrolled 12 healthy volunteers with no history of vascular or cardiac disease. One subject (23 years old female) was excluded as we were unable to obtain the plethysmograph signals on the toe. Demographics, baseline characteristics, values of PWV_ΔPAT Toe-Finger_, ΔPWV_Standing-Supine_, ΔET _Toe-Finger_, ET index and the group assignment based on the ET index of individual subjects are presented in Table 1.

**Table 1:** Demographics, baseline characteristics, values of PWV_ΔPAT Toe-Finger_, ΔPWV_Standing-Supine_, ΔET _Toe-Finger_, ET index and group assignment based on ET index of the subjects.

	Age	Gender	BMI	MAP	HR	PWV_ΔPAT_	ΔPWV	ΔET	ET index	Group
		M:male				Toe-Finger	Toe-Finger	Toe-Finger	Toe-Finger	
		F:female		Supine	Supine	Supine	Standing-Supine	Supine	Supine-Standing	
No.	(y.o.)		(kg/m^2^)	(mmHg)	(bpm)	(m/s)	(m/s)	(ms)	(ms)	
1	50	M	26.3	82	62	6.16	1.52	59.2	2.5	1
2	39	M	31.9	91	77	7.17	3.58	34.7	-0.6	1
3	36	F	20.2	86	57	8.06	3.02	24.2	15.5	1
4	41	M	27.1	82	61	7.02	19.16	30.7	40.5	1
5	29	M	23.2	81	56	7.38	3.88	32.0	31.5	1
6	31	M	26.0	80	76	6.44	3.50	69.8	51.3	2
7	36	F	21.6	67	57	5.28	-0.88	31.3	59.4	2
8	26	F	20.8	68	78	6.20	2.39	97.6	64.0	2
9	23	F	20.6	65	60	3.90	8.89	108.2	79.9	2
10	26	M	30.1	81	74	6.45	4.54	78.2	65.7	2
11	23	M	23.0	82	84	7.48	5.28	57.4	45.6	2
Mean	33		24.6	79	67	6.50	4.99	56.7	41.4	
SD	9		4.0	8	10	1.16	5.28	29.1	26.6	

BMI: body mass index; MAP: mean arterial pressure; HR: heart rate; PWV: pulse wave velocity; PAT: pulse arrival time; ET: ejection time.

S1 Fig is a schematic representation of the simultaneously recorded electrocardiogram and plethysmograph waveforms at the ear, finger and toe in the standing, sitting and supine positions. The dots on the ECG waveforms indicate the peak of the R wave, the dots on the plethysmograph waveforms indicate the beginning of the upstroke of the pulse waveform and the dicrotic notch. The ETs are shaded in gray between the start of the upstroke and the dicrotic notch on the plethysmograph waveform. The waveform presented in the figure was obtained from a single subject from each location but the duration of the ET and the dots represent average times, as calculated from all subjects.

**Fig 1:**
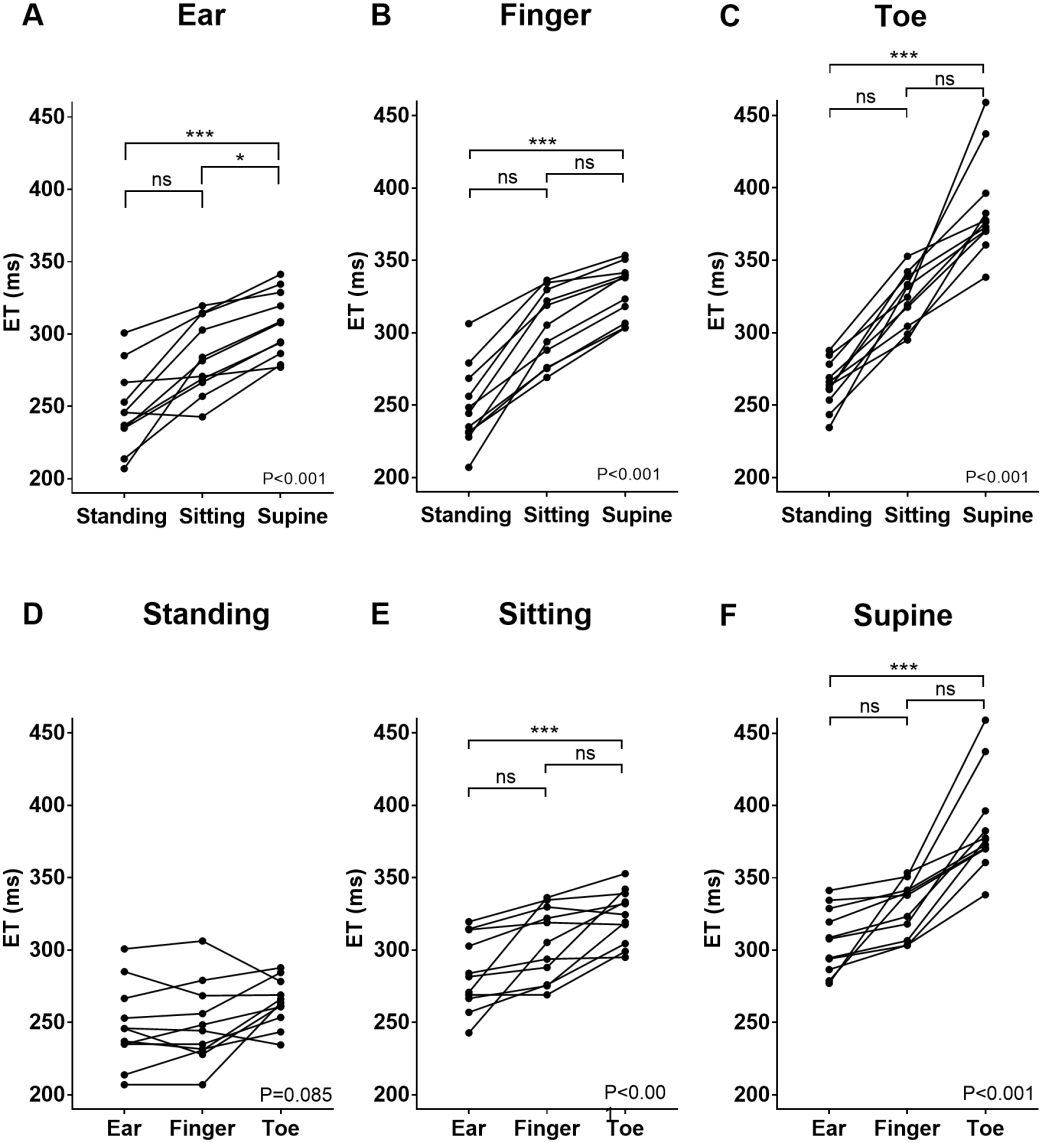
Effect of position and peripheral location on the measured ET. Each plot indicates the mean ET calculated from each individual subject. (A) ETs measured at the ear. (B) ETs measured at the finger. (C) ETs measured at the toe. (D) ETs measured in the standing position. (E) ETs measured in the sitting position. (F) ETs measured in the supine position. ET: ejection time, ns: not significant, *: P < 0.05, ***: P < 0.001.

All measurements tabulated are shown in Table 2. The RR interval was shorter (faster HR) in the standing compared to the sitting and supine position (standing vs sitting: P = 0.017, standing vs supine: P<0.001, sitting vs supine: P>0.99). The ET was shorter in the standing compared to the supine position—irrespective of the location (standing vs supine: P<0.001 at all locations) (Fig 1A–1C). The comparison between the ETs measured at different locations in the standing position didn’t reach statistical significance (P = 0.085) (Fig 1D). The ET was shorter at the ear compared to the toe in the sitting and supine positions (P<0.001 in both positions) (Fig 1E and 1F).

**Table 2:** Mean±SD of RR interval, ET, ΔET, ΔPAT, ΔDAT and PWV in 3 positions.

	Standing	Sitting	Supine
**RR interval (ms)**	747.9 ± 154.0	866.0 ± 149.9	907.3 ± 135.1
**ET (ms)**			
Ear	247.7 ± 28.0	283.9 ± 25.7	306.5 ± 22.4
Finger	248.7 ± 27.7	304.5 ± 25.3	329.0 ± 18.7
Toe	263.9 ± 16.2	323.6 ± 18.6	385.6 ± 34.4
**ΔET (ms)**			
Toe—Ear	16.2 ± 25.9	39.7 ± 29.5	79.1 ± 36.3
Toe—Finger	15.3 ± 23.3	19.1 ± 19.4	56.7 ± 29.1
Finger—Ear	0.9 ± 11.3	20.6 ± 22.3	22.5 ± 23.5
**ΔPAT (ms)**			
Toe—Ear	135.7 ± 23.4	141.6 ± 20.0	191.0 ± 17.9
Toe—Finger	65.4 ± 26.9	62.6 ±12.5	101.9 ± 20.9
Finger—Ear	70.3 ± 10.4	79.0 ± 12.1	89.1 ± 10.6
**ΔDAT (ms)**			
Toe—Ear	147.2 ± 36.5	180.3 ± 42.8	269.7 ± 49.9
Toe—Finger	76.5 ± 33.5	80.5 ± 29.1	158.3 ± 47.2
Finger—Ear	70.7 ± 18.2	99.8 ± 24.2	111.3 ± 29.2
**PWV**_**ΔPAT**_ **(m/s)**			
Toe—Ear	9.59 ± 1.97	9.05 ± 1.26	6.65 ± 0.64
Toe—Finger	11.49 ± 5.44	10.64 ± 2.29	6.50 ± 1.16
Finger—Ear	8.98 ± 1.37	7.98 ± 1.10	7.03 ± 0.83
**PWV**_**ΔDAT**_ **(m/s)**			
Toe—Ear	9.16 ± 2.85	7.36 ± 1.76	4.81 ± 0.85
Toe—Finger	11.16 ± 9.35	9.01 ± 3.40	4.34 ± 1.08
Finger—Ear	9.24 ± 2.09	6.53 ± 1.51	5.87 ± 1.29

SD: standard deviation, ET: ejection time, PAT: pulse arrival time, DAT: dicrotic notch arrival time, PWV: pulse wave velocity.

**Fig 2:**
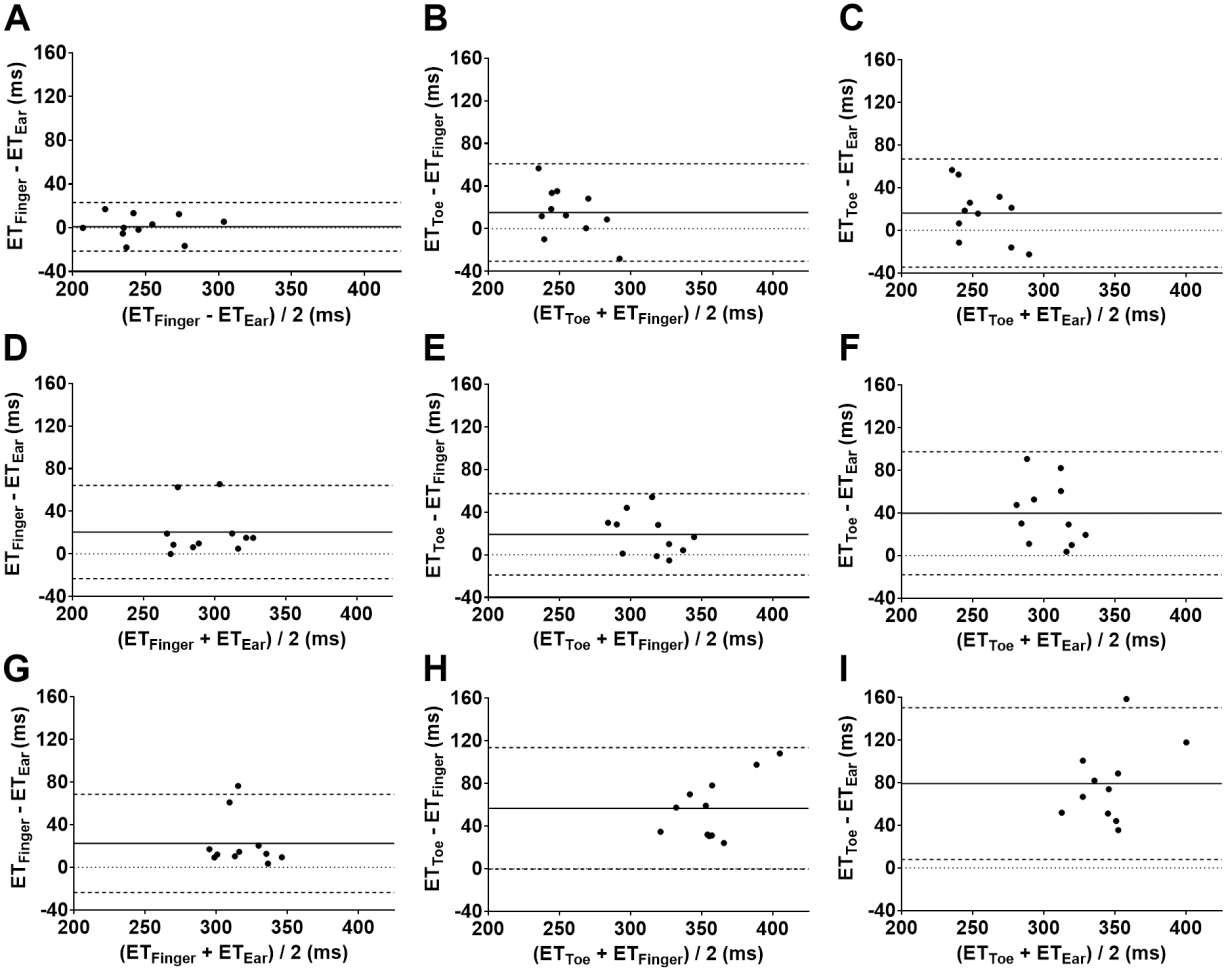
Bland-Altman plots of the difference between ETs measured at two peripheral locations. Bland Altman plots showing the difference between ET at the finger and ET at the ear (A)(D)(G), between ET at the toe and ET at the finger (B)(E)(H), and between ET at the toe and ET at the ear (C)(F)(I). Each ET was measured in the standing (A)(B)(C), sitting (D)(E)(F), and supine (G)(H)(I) position. The solid line indicates the mean of difference, and the dashed line indicates the 95% limits of agreement. The difference of ETs between each pair of locations are also shown in Table 2 (ΔET). ET: ejection time; SD: standard deviation.

The ΔET was shorter in standing compared to the supine position at all locations (standing vs supine: P<0.001 for tor-ear, P = 0.004 for toe-finger, P = 0.009 for finger-ear). The ΔETs in the standing and the ΔETs in the sitting position were not significantly different at toe-ear or toe-finger (P>0.99 at both locations), however, the ΔET in the standing position was shorter compared to sitting for finger-ear (P = 0.017). A Bland-Altman analysis comparing ET at different locations (figure vs ear, toe vs finger, toe vs ear) in different positions (standing, sitting, and supine) is depicted in Fig 2. The difference of ETs measured at two different locations are shortest in the standing position and longest in the supine position.

PWV_ΔPAT_ was faster in the standing compared to the supine position irrespective of the pair of locations (standing vs supine: P<0.001 for toe-ear and finger-ear, P = 0.004 for toe-finger). PWV_ΔPAT_ in standing and PWV_ΔPAT_ in the sitting position were not significantly different at all pairs of locations.

PWV_ΔDAT_ was faster in the standing compared to the supine position irrespective of the pair of locations (standing vs supine: P<0.001 for all pairs of locations). PWV_ΔDAT_ in the standing and PWV_ΔDAT_ in the sitting position were not significantly different for toe-ear and toe-finger, however, PWV_ΔDAT_ while standing was faster compared to the sitting position for finger-ear (P = 0.009).

ΔET between all pairs of locations correlated with the corresponding ΔPAT and ΔDAT (S2 Fig). The R^2^ was higher for ΔDAT compared to ΔPAT for all pairs of locations. The best correlation was observed for the difference in ETs between the toe and ear (ΔET_Toe-Ear_) compared to the difference in DAT (ΔDAT_Toe-Ear_) with R^2^ = 0.86. ΔDAT showed a significant correlation with ΔPAT between all locations. (R^2^ = 0.80, P<0.001 for toe-ear, R^2^ = 0.80, P<0.001 for toe-finger, R^2^ = 0.52, P<0.001 for finger-ear).

**Fig 3:**
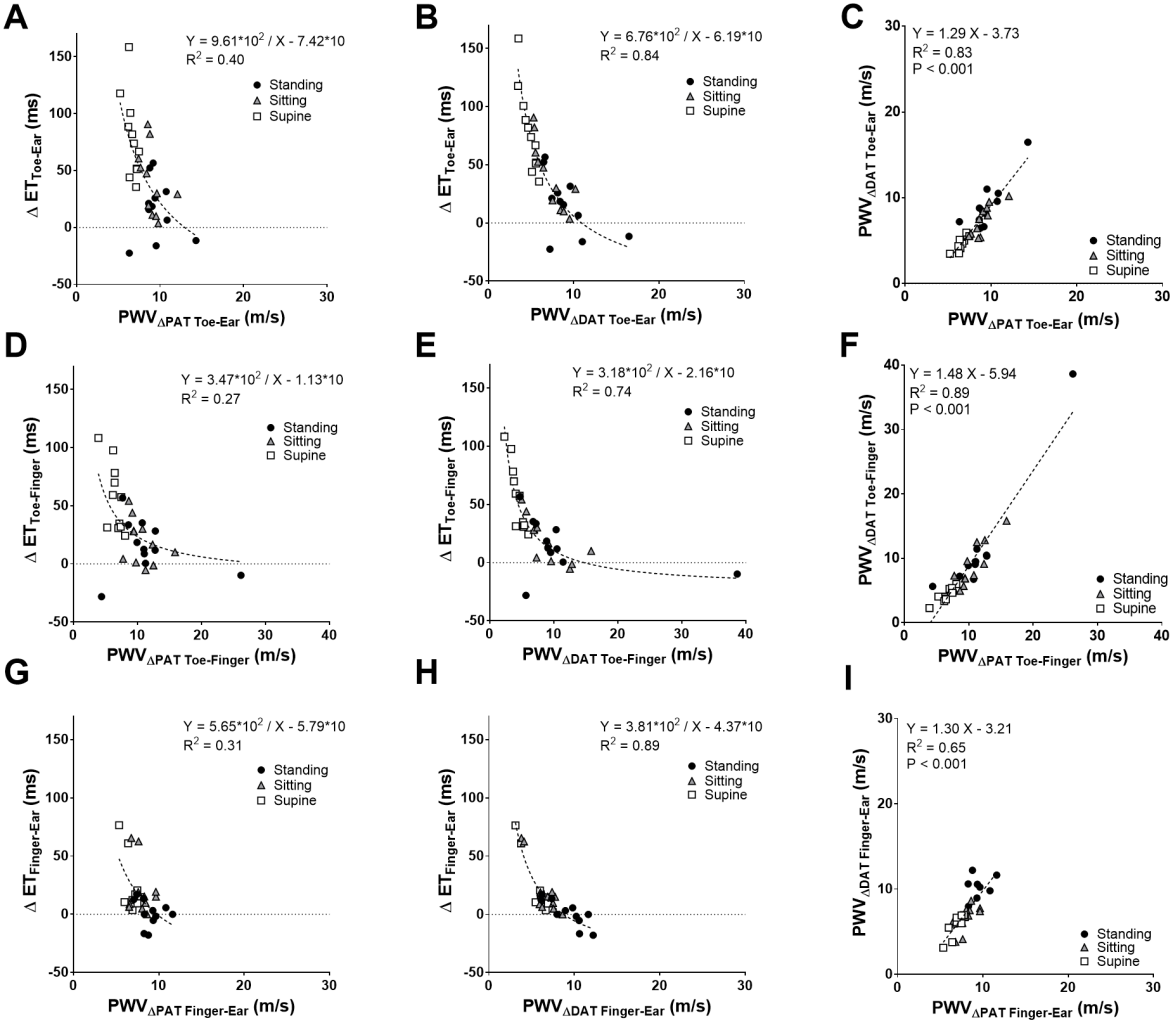
Relationship between ΔET, PWV_ΔPAT_, and PWV_ΔDAT_. The relationship between ΔET and PWV_ΔPAT_ (A, D, G), between ΔET and PWV_ΔDAT_ (B, E, H), between PWV_ΔDAT_ and PWV_ΔPAT_ (C, F, I) for each pair of locations (Toe-Ear: A, B, C; Toe-Finger: D, E, F; Finger-Ear: G, H, I). Each individual subject has three dots on each graph which indicate mean values derived from the standing (black circle), sitting (gray triangle) and supine (white square) positions. The dashed line indicates the hyperbolic or linear regression line. The regression equation and the coefficient of determination (R^2^) are presented in each graph. ET: ejection time; PWV: pulse wave velocity; PAT: pulse arrival time; DAT: dicrotic notch arrival time.

ΔET decreased exponentially with increasing PWV (either based on ΔPAT or ΔDAT) for each pair of locations (Fig 3A, 3B, 3D, 3E, 3G and 3H). R^2^ was higher for PWV_ΔDAT_ compared to PWV_ΔPAT_ in all pairs of locations. PWV_ΔDAT_ correlated well with PWV_ΔPAT_ with a R^2^ = 0.89 for toe-finger (Fig 2F).

**Fig 4:**
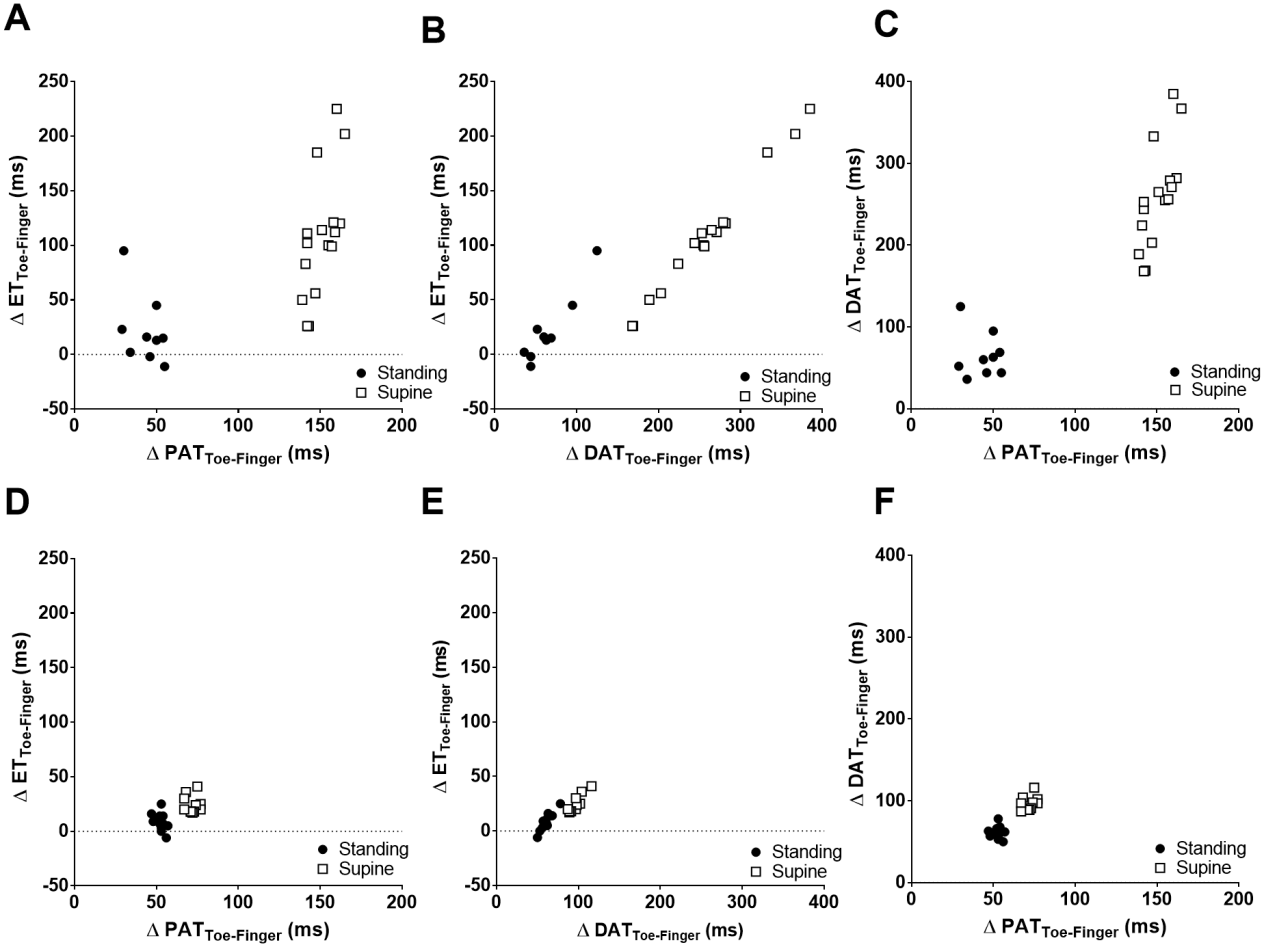
Relationship between ΔET_Toe-Finger_, ΔPAT_Toe-Finger_ and ΔDAT_Toe-Finger_ in an individual subject. (A), (B), and (C) Measurements derived from a 23 year old female with the most compliant vasculature as evident by the lowest PWV_ΔPAT Toe-Finger_ in the supine position. (D), (E), and (F) Measurements derived from a 36 year old female with the least compliant vasculature as evident by highest PWV_ΔPAT Toe-Finger_ in the supine position. Each dot on the graph was derived from a single heartbeat in the standing (black circles) and supine (white squares) positions. (A) and (D) Scatter plot of ΔET_Toe-Finger_ and ΔPAT_Toe-Finger_. (B) and (E) Scatter plot of ΔET_Toe-Finger_ and ΔDAT_Toe-Finger_. (C) and (F) Scatter plot of ΔDAT_Toe-Finger_ and ΔPAT_Toe-Finger_. ET: ejection time; PAT: pulse arrival time; DAT: dicrotic notch arrival time, PWV: pulse wave velocity.

We depict the relationships between ΔET_Toe-Finger_, ΔPAT_Toe-Finger_, and ΔDAT_Toe-Finger_ in two representative subjects in Fig 4. One subject had the most compliant vasculature and the other the least compliant vasculature, based on PWV_ΔPAT Toe-Finger_ in the supine position. Fig 4A–4C show the relationships between ΔET_Toe-Finger_, ΔPAT_Toe-Finger_, and ΔDAT_Toe-Finger_ as derived from the subject with the most compliant vasculature (PWV_ΔPAT Toe-Finger_ of 3.90 m/s in the supine and 12.79 m/s in the standing position in a 23 years old female). ΔET_Toe-Finger_, ΔPAT_Toe-Finger_, and ΔDAT_Toe-Finger_ varied with changing positions, with ΔET_Toe-Finger_ and ΔDAT_Toe-Finger_ varying more than ΔPAT_Toe-Finger_. The average of ΔET_Toe-Finger_ was 28.3 ms while standing and increased 3.8 times in the supine position to 108.2 ms in this young female with a compliant vasculature. The difference in absolute values between ΔET_Toe-Finger_ supine and standing (ET index) was 79.9 ms.

The relationship between ΔET_Toe-Finger_, ΔPAT_Toe-Finger_, and ΔDAT_Toe-Finger_ derived from the subject with the least compliant vasculature as evident by highest PWV_ΔPAT Toe-Finger_ in the supine position (PWV_ΔPAT Toe-Finger_ of 8.06 m/s in supine and 11.08 m/s in standing in a 36 years old female), are depicted in Fig 4D–4F. The variation of ΔET_Toe-Finger_, ΔPAT_Toe-Finger_, and ΔDAT_Toe-Finger_ were small compared to the subject with compliant vasculature. The average ΔET_Toe-Finger_ was 8.7 ms when standing and increased 2.8 times in the supine position to 24.2 ms. The difference in absolute values between ΔET_Toe-Finger_ supine and standing (ET index) was 15.5 ms, which is 5.2 times less than the 79.9 ms observed in the subject with a more compliant vasculature.

**Fig 5:**
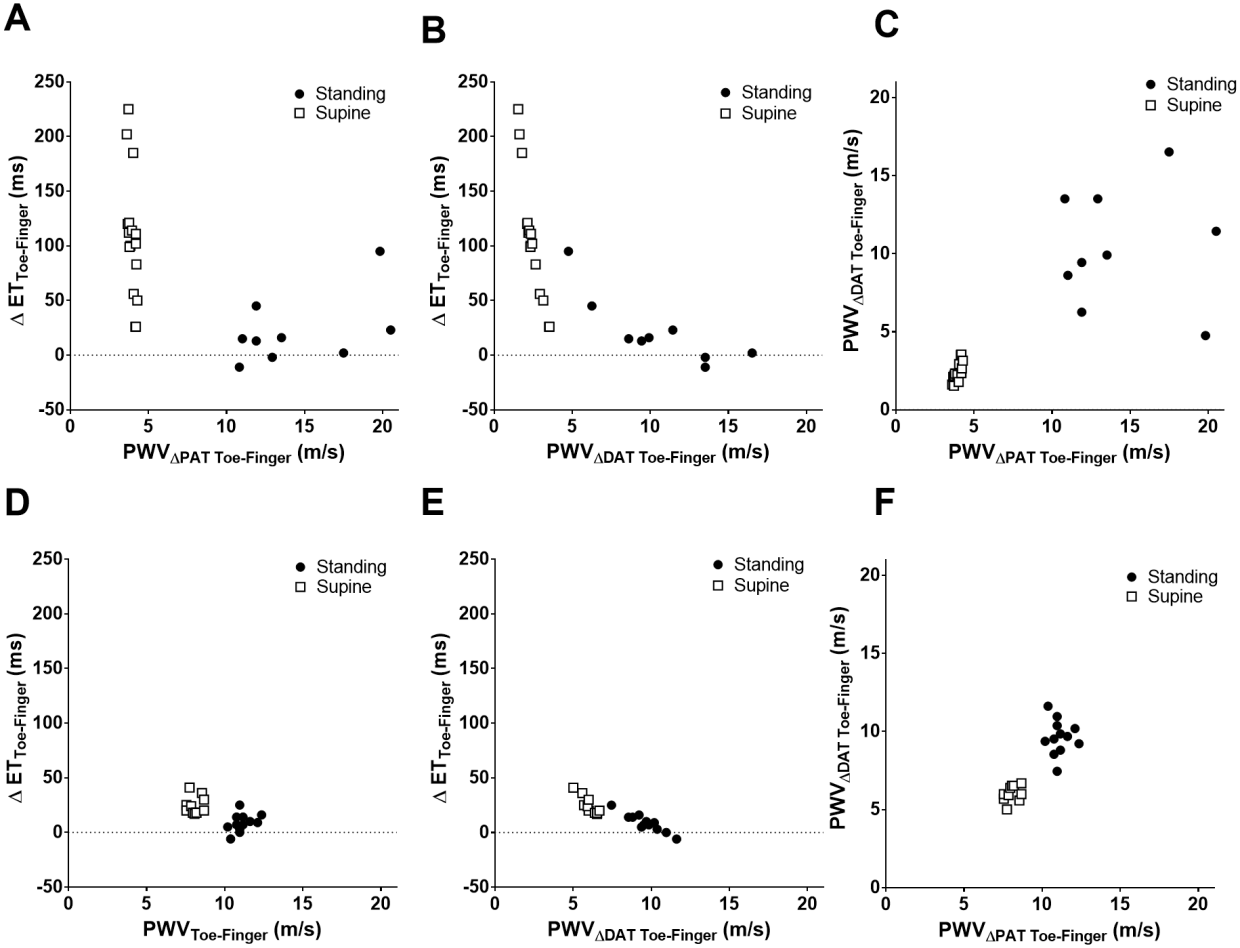
Relationship between ΔET_Toe-Finger_, PWV_ΔPAT Toe-Finger_, and PWV_ΔDAT Toe-Finger_ in individual subject. (A), (B), and (C) Measurements derived from a 23 year old female with the most compliant vasculature as evident by the lowest PWV_ΔPAT Toe-Finger_ in the supine position. (D), (E), and (F) Measurements derived from a 36 year old female with the least compliant vasculature as evident by the highest PWV_ΔPAT Toe-Finger_ in the supine position. Each dot on the graph was derived from a single heartbeat in the standing (black circles) and supine (white squares) position. (A) and (D) Scatter plot of ΔET_Toe-Finger_ and PWV_ΔPAT Toe-Finger_. (B) and (E) Scatter plot of ΔET_Toe-Finger_ and PWV_ΔDAT Toe-Finger_. (C) and (F) Scatter plot of PWV_ΔDAT Toe-Finger_ and PWV_ΔPAT Toe-Finger_. ET: ejection time; PWV: pulse wave velocity; PAT: pulse arrival time; DAT: dicrotic notch arrival time.

We compared the relationships between ΔET_Toe-Finger_, PWV_ΔPAT Toe-Finger_, and PWV_ΔDAT Toe-Finger_ in the same two subjects (most and least compliant vasculature based on PWV_ΔPAT Toe-Finger_ in the supine position) (Fig 5). We observed a significant variability in ΔET_Toe-Finger_ in the supine position in the subject with the most compliant vasculature (Fig 5A and 5B) compared to the subject with the least compliant vasculature (Fig 5D and 5E). Similarly we observed significant variability in PWV (both PWV_ΔPAT Toe-Finger_ and PWV_ΔDAT Toe-Finger_) in standing position in the subject with the most compliant vasculature (Fig 5A–5C).

**Fig 6:**
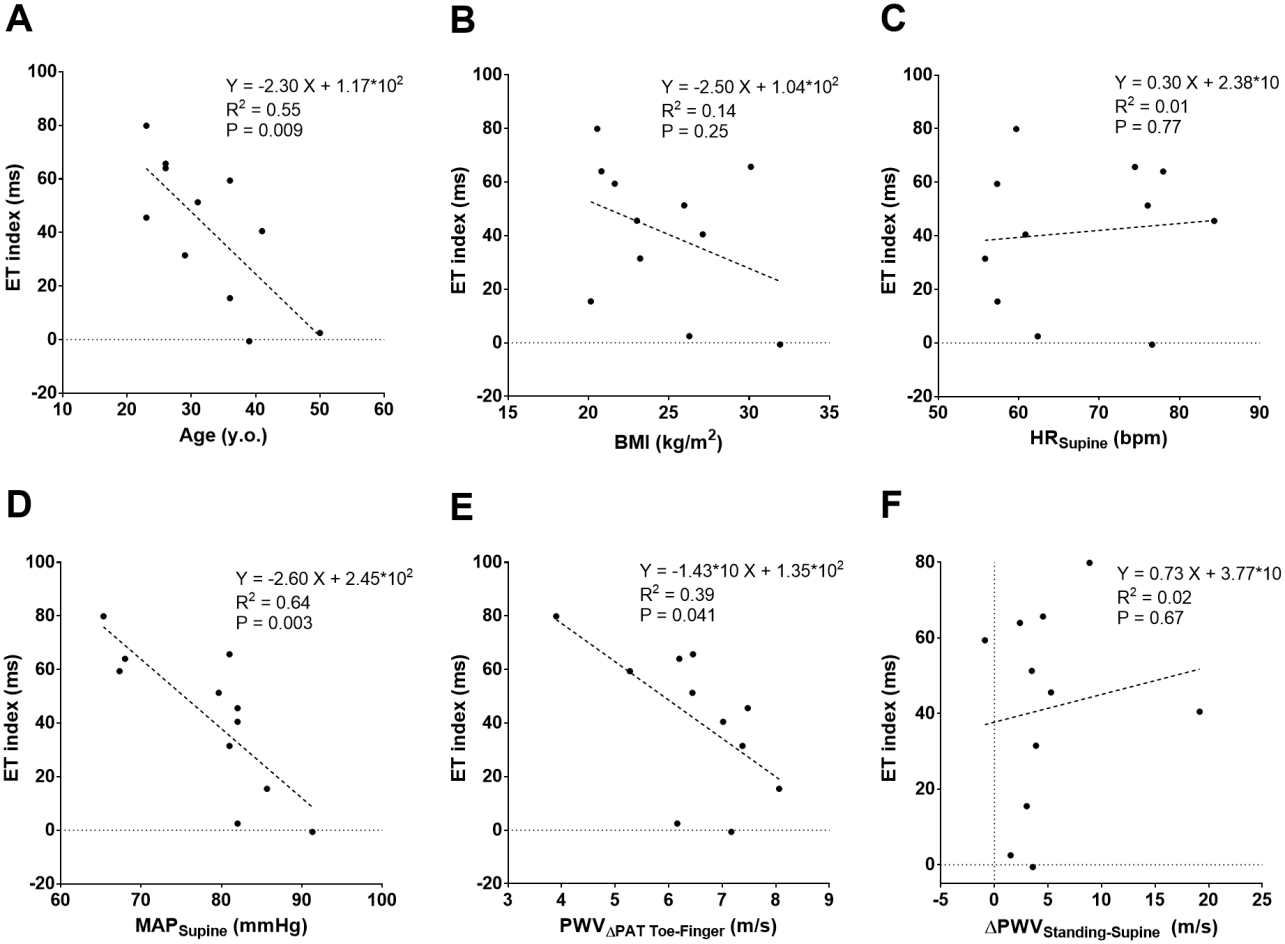
The relationship between the ET index and age, BMI, HR, MAP, PWV_ΔPAT Toe-Finger_, and ΔPWV_Standing-Supine_. The ET index was calculated by subtracting the mean ΔET_Toe-Finger_ in the standing position from the mean ΔET_Toe-Finger_ in the supine position for each individual subject. The dashed line indicates the simple linear regression line. The regression equation, the coefficient of determination (R^2^), and P value are presented. (A) ET index as a function of age. (B) ET index as a function of BMI. (C) ET index as a function of HR measured in the supine position. (D) ET index as a function of MAP measured in the supine position. (E) The ET index as a function of PWV_ΔPAT Toe-Finger_ in the supine position. (F) The ET index as a function of ΔPWV_Standing-Supine_. ΔPWV_Standing-Supine_ was calculated as the difference between PWV_ΔPAT Toe-Finger_ in the supine and standing position. ET: ejection time; BMI: body mass index; HR: heart rate; MAP: mean arterial pressure; PWV: pulse wave velocity.

Given that there was a significant change in the ET index between the two subjects with the most and least compliant vasculatures, we explored this parameter, ET index, in all subjects. Fig 6 depicts the relationship between the ET index and age (Fig 6A), body mass index (BMI) (Fig 6B), HR measured in the supine position (Fig 6C), MAP measured in the supine position (Fig 6D), PWV_ΔPAT Toe-Finger_ measured in the supine position (Fig 6E), and the difference between PWV_ΔPAT Toe-Finger_ in the supine and standing position (ΔPWV_Standing-Supine_, Fig 6F). Each individual subject represents a dot on the graph. The ET index correlated significantly with age, MAP, and PWV_ΔPAT Toe-Finger_ such that increased age, MAP, and PWV_ΔPAT Toe-Finger_ were associated with decreased ET index. However, ET index did not correlate with BMI, HR, or ΔPWV_Standing-Supine_.

Performing a GLM analysis revealed that younger age and lower MAP were significantly associated with a higher ET index (coefficient: -1.75, p<0.001; coefficient: -3.16, p = 0.001, respectively). However, PWV_ΔPAT Toe-Finger_ was not significantly associated with the ET index (p = 0.40) (Table 3A).

**Table 3:** GLM analysis.

Parameter	Coefficient	95% confidence interval	p value
**(A) Dependent variable: ET index**			
(Intercept)	273.15	194.77–351.54	<0.001
**Age**	-1.75	-2.57–-0.94	**<0.001**
Gender	3.51	-13.63–20.65	0.69
BMI	2.58	-0.17–5.33	0.066
**MAP supine**	-3.16	-5.02–-1.31	**0.001**
HR supine	-0.30	-0.96–0.36	0.37
PWV _ΔPAT Toe-Finger_	4.49	-5.95–14.92	0.40
**(B) Dependent variable: ΔET**_**Toe-Finger**_			
(Intercept)	112.89	-19.33–245.12	0.094
Age	-0.75	-2.13–0.62	0.28
Gender	4.32	-24.59–33.23	0.77
BMI	-1.90	-6.54–2.74	0.42
MAP supine	0.75	-2.38–3.87	0.64
**HR supine**	1.37	0.25–2.48	**0.016**
**PWV**_**ΔPAT Toe-Finger**_	-21.29	-38.88–-3.69	**0.018**

GLM: generalized linear model; ET: ejection time; BMI: body mass index; HR: heart rate; MAP: mean arterial pressure; PWV: pulse wave velocity; PAT: pulse arrival time.

Then we investigated if the effect of age, BMI, HR, MAP, PWV_ΔPAT Toe-Finger_, and ΔPWV_Standing-Supine_ on ΔET_Toe-Finger_ in the supine position were similar to the ET index. R^2^ was lower for age vs ΔET_Toe-Finger_ (R^2^ = 0.30, P = 0.08), and for MAP vs ΔET_Toe-Finger_ (R^2^ = 0.35, P = 0.06) and higher for PWV_ΔPAT Toe-Finger_ vs ΔET_Toe-Finger_ (R^2^ = 0.42, P = 0.03). Similar to the ET index, BMI, HR, and ΔPWV_Standing-Supine_ were not correlated with ΔET_Toe-Finger_.

The GLM analysis revealed further that increased HR and decreased PWV_ΔPAT Toe-Finger_ were significantly associated with an increased ΔET_Toe-Finger_ (coefficient: 1.37, p = 0.016; coefficient: -21.29, p = 0.018, respectively) (Table 3B).

We also investigated the effect of age, BMI, HR, MAP, and PWV_ΔPAT Toe-Finger_ on ΔPWV_Standing-Supine_. All variables were not correlated with ΔPWV_Standing-Supine_ (S3 Fig).

**Fig 7:**
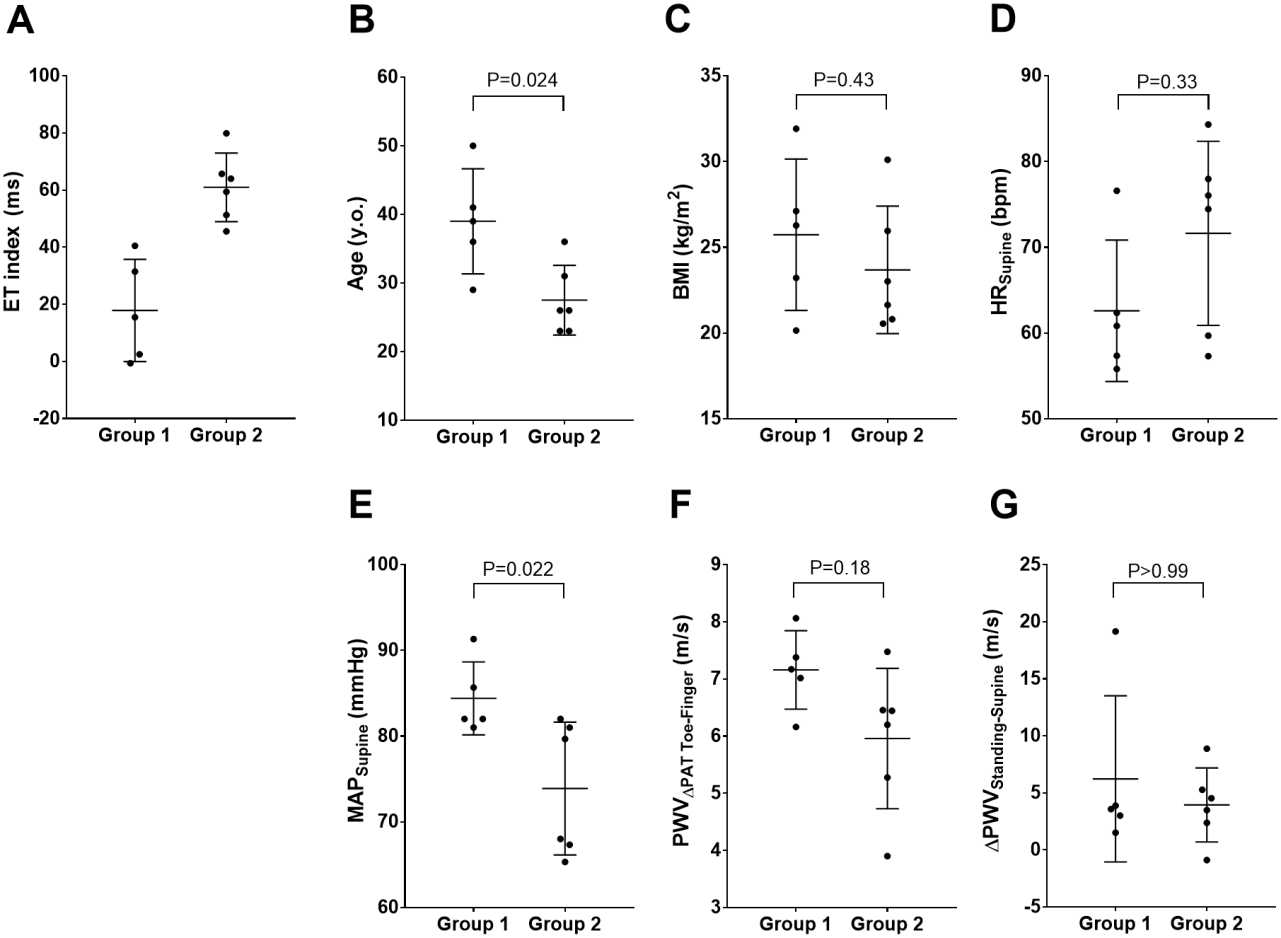
The comparison between group 1 and group 2. Subjects were divided into two groups according to their percentile rank of the ET index: group 1 had low ET index and group 2 had high ET index (A). Each graph showing the distribution of age (B), BMI (C), HR in supine (D), MAP supine (E), PWV_ΔPAT Toe-Finger_ supine (F), and ΔPWV_Standing-Supine_ (G) in group 1 and group 2. Each graph represents the individual data points and superimposes a horizontal line at the arithmetic mean; error bars showing plus and minus one SD. ET: ejection time; BMI: body mass index; HR: heart rate; MAP: mean arterial pressure; PWV: pulse wave velocity; SD: standard deviation.

To investigate if the ET index could differentiate a more compliant from a less compliant vasculature we divided all subjects into two groups according to their percentile rank of the ET index: group 1 (below 50% of ET index, mean ±SD of 17.9±17.9ms) and group 2 (above 50% of ET index, mean ±SD of 61.0±12.0ms) (Fig 7A). The group assignment is also shown in Table 1. The number of males and females were not significantly different between the two groups (P = 0.55). Subjects in group 2 with a higher ET index were younger (28±5 years vs 39±8 years, P = 0.024) (Fig 7B), had lower MAPs (74±8 mmHg vs 84±4 mmHg, P = 0.022) (Fig 7E), and tended to have lower PWV_ΔPAT Toe-Finger_ (5.96±1.23 m/s vs 7.16±0.69 m/s, P = 0.18,) (Fig 7F) compared to the subjects in group 1 with a lower ET index. BMI, HR, and ΔPWV_Standing-Supine_ were not different between the two groups (P = 0.43, P = 0.33, and P>0.99 respectively) (Fig 7C, 7D and 7G).

Lastly, we divided all subjects into two groups according to their percentile rank of the ΔET_Toe-Finger_ in the supine position to investigate if ΔET_Toe-Finger_ could differentiate a more compliant from a less compliant vasculature similar to the ET index (S4A Fig). We found that subjects in group 2 with a higher ΔET_Toe-Finger_ tended to be younger (30±10 years vs 36±5 years, P = 0.11) (S4B Fig), have lower MAPs (76±8 mmHg vs 81±9 mmHg, P = 0.26) (S4E Fig), and lower PWV_ΔPAT Toe-Finger_ (6.12±1.18 m/s vs 6.98±1.03 m/s, P = 0.25) (S4F Fig) compared to subjects in group 1 with a lower ΔET_Toe-Finger_. However, this didn’t reach statistical significance.

## Discussion

In this study we found that ejection times at the ear, finger, and toe are different for the same heartbeat and become longer as the measurement site distance from the heart increases. Furthermore, the ejection times at individual measurement sites are different in different positions: longest in the supine position and shortest in the standing position. As a result, the difference between ET at different locations (ΔET) for the same heartbeat is maximal for the toe versus the ear in the supine position. This difference becomes smallest in the standing position for all pairs of locations. Moreover we show that this difference in ETs (ΔET) is distinct in different subjects and correlates with age, blood pressure, PWV, and the corresponding pulse arrival times. Consistent with our hypothesis we found that higher values for ΔET in the supine position are generally observed in younger patients with lower MAPs and lower PWVs indicating that the prolongation of the ET at more distal sites (e.g. the toe) compared to more proximal sites (e.g. the ear or finger) might represent novel index of vascular properties.

Our results add further knowledge to two recent studies which demonstrated that LVET correlates with cf-PWV [17,18]. They speculated that the initial speed of the resultant pressure wave is mainly determined by the velocity of myocardial shortening which in turn is a function of myocardial wall movement and the duration of left ventricular ejection. Thus, an increase in LVET is correlated with a decrease in cf-PWV [17]. Since our study focused on peripherally measured ETs and we calculated ΔET for the same heartbeat, this variable is independent from the true ET (LVET), making our study substantially different from previous studies.

We report a PWV_ΔPAT Toe-Finger_ as 6.5 ± 1.2m/s which is 1.5 m/s higher than previously reported MRI based aortic PWV values [19]. We believe that the main reason for this is that large vessels (e.g. aorta) have lower PWVs compared to the smaller diameter peripheral vessels [20]. PWV_ΔPAT Toe-Finger_ is based on a vascular path length which includes both large and small vessels (central aorta and peripheral arteries). That is why the presented PWV is higher than PWV in aorta as reported in MRI based studies. Furthermore, a large multi-center study on normal carotid-femoral PWV as measured by tonometry demonstrated values of 6.1 m/s in healthy less than 30 years old individuals and 6.6 m/s in 30 to 40 years old individuals [21]. Our results are therefore comparable to previously reported carotid-femoral PWV values.

Our group has shown previously that postural changes significantly affect PAT to different locations, most likely due to changes in the vasculature’s distending pressure and wall tension associated with changing positions from supine to sitting and standing [12]. Our group also reported that a peripherally measured ET derived from the radial arterial waveform is longer than the centrally measured ET derived from CW Doppler through the aortic valve [11]. We showed that prolongation of the ET at a peripheral site becomes more pronounced at lower BPs and PWVs suggesting a modulating effect of the vasculature on the central ventricular ejection time [11]. Similarly we believe that the observed difference between ETs at two different peripheral locations appears to be due to the modulating effect of the vasculature on the original central ET and represents intrinsic vascular properties.

Given that the ΔET is longest in the supine position and shortest when standing we looked at the difference (ET index) in individual subjects as a potential marker to distinguish a compliant from a stiff vasculature. By definition, a compliant vasculature is more distensible than a stiff vasculature, the influence of postural changes on ET prolongation should be larger in subjects with a compliant vasculature compared to those with a stiff vasculature. There should also be a significant prolongation of the ET measured at the toe in the supine position if the vasculature is compliant. Indeed both ΔET and ET index are longer in young people with lower blood pressure and lower PWV. Interestingly change in PWV between supine and standing (ΔPWV) does not correlate with either the ET index or ΔET. In contrast to the ET index, ΔPWV_Standing-Supine_ does not correlate with age, MAP nor PWV, suggesting that the ET index better reflects intrinsic vascular properties. Moreover, the ΔPWV_Standing-Supine_ difference between the most compliant (8.89 m/s) and stiffest (3.88 m/s) subjects was only differed by a factor of 2, whereas the ET index (79.9 ms in compliant and 15.5 ms in stiff) differed by a factor of 5 for the same subjects indicating that our novel parameter might potentially be more sensitive to assess arterial stiffness, and vascular properties.

Given that the ΔET in the standing position was small in most subjects and the ET index is different between ΔET in the supine vs standing position, we investigated if ΔET in the supine position alone will have comparable to the ET index correlation with age, MAP, and PWV. In fact ΔET in the supine position had a better correlation with PWV_ΔPAT Toe-Finger_ than ET index, however, worse correlation with age and MAP.

We also found that ΔET correlates with the corresponding ΔPAT and PWV. Both ΔPAT_Toe-Finger_ and PWV_ΔPAT Toe-Finger_ have been reported as a good alternatives for the measurement of arterial stiffness [16,22]. Alivon et al. demonstrated that PWV_ΔPAT Toe-Finger_ is significantly correlated with cf-PWV which can be considered as the current gold standard of arterial stiffness [16].

These findings suggest that both ΔET and ET index are potential novel markers of vascular properties and support the notion that intra-vascular hydrostatic changes associated with changing position from supine to standing corresponds to significant changes in wall tension and vascular properties. Moreover ΔET and the ET index appear to be more sensitive markers compared to changes in PWV between the supine and standing positions.

Our study has several limitations. We used plethysmography with ECG gating for pulse wave detection. This technique estimates global stiffness along the aortic-peripheral vessel tract rather than stiffness in a specific portion of the vascular tree (e.g. central aorta). The study was conducted on only adult self-reported healthy volunteers. The number of subjects included in the study was small. However it was more than two times higher compared to the calculated sample size. The plethysmograph signal is known to be susceptible to various external or internal influences, including motion artifact, vascular tone and contacting force [23,24]. One subject who met all the inclusion criteria had to be excluded from the study because of the poor quality of plethysmograph trace. We used PWV based on PAT to toe and finger rather than cf-PWV. Also, we assumed the vascular path length from a body surface measurement, namely the distance from sternal notch to each peripheral location. This might have introduced bias in all PWV measurements. Some of these limitations can be mitigated by MRI measurement, which provides a precise measurement of vascular path length and arterial stiffness in the central aorta, albeit with loss of temporal resolution [25].

## Conclusions

ETs are different at different peripheral locations for the same heartbeat: the further the site of measurement from the heart, the longer the ET. Consisting with our hypotheses, prolongation of the ET at a distal site compared to a proximal site of measurement is much more pronounced in subjects with more compliant vasculature and in the supine position. The ET difference between two peripheral locations (ΔET) correlates with the corresponding PWV, MAP, and age suggesting that the ET difference represents the underlying vascular properties. Similarly, the difference between ΔET in the supine and standing position (ET index) represents the dynamic hydrostatic blood pressure change that correlates with the corresponding PWV, MAP, and age suggesting that the ET difference represents the underlying vascular properties. We propose ΔET as a potential novel real-time continuous and non-invasive parameter of vascular properties, and the ET index as a potential non-invasive parameter of vascular reactivity. Given that this study is an exploratory pilot study, confirmatory evaluations in an adequately large population of subjects of different ages are needed to validate these results. ΔET could be easily extracted from real-time non-invasive monitors of plethysmograph waveforms. The ET index could be quickly and easily obtained at an office setting for risk prediction and stratification. We envision that the ET based approach could be utilized for the assessment of vascular function in patients with hypertension to stratify them and to assess patient responses to different treatment options. Again, further studies are needed to investigate the utilities of ΔET and the ET index in patient monitoring and risk stratification in various clinical populations.

## Supporting information

S1 Fig Schematic representation of the electrocardiogram and plethysmograph waveforms at the ear, finger and toe in the standing, sitting and supine positions. The dots on the electrocardiogram waveforms indicate the peak of R wave, the dots on the plethysmograph waveforms indicate the beginning of the upstroke and dicrotic notch. ET defined between the start of upstroke and dicrotic notch on plethysmograph waveform is shaded gray. The waveform is representative from each location but the duration of ET and dots represent average time calculated from all subjects. ET: ejection time.
(TIF)

S2 Fig Relationship between ΔET and ΔPAT (A, D, G), between ΔET and ΔDAT (B, E, H), between ΔDAT and ΔPAT (C, F, I) for each pair of locations (Toe-Ear: A, B, C, Toe-Finger: D, E, F, Finger-Ear: G, H, I). Each individual subject has three dots on the each graph which indicate mean values derived from the standing (black circles), sitting (gray triangles) and supine (white squares) positions. The dashed line indicates the linear regression line. The regression equation, the coefficient of determination (R^2^), and P value are presented. ET: ejection time; PAT: pulse arrival time; DAT: dicrotic notch arrival time.
(TIF)

S3 Fig ΔPWV_Standing-Supine_ and age, BMI, HR, MAP, PWV_ΔPAT Toe-Finger_. (A) ΔPWV_Standing-Supine_ as a function of age. (B) ΔPWV_Standing-Supine_ as a function of BMI. (C) ΔPWV_Standing-Supine_ as a function of HR measured in supine position. (D) ΔPWV_Standing-Supine_ as a function of MAP measured in supine position. (E) ΔPWV_Standing-Supine_ as a function of PWV_ΔPAT Toe-Finger_ in supine position. ΔPWV_Standing-Supine_ was calculated by subtracting the mean of PWV_ΔPAT Toe-Finger_ in supine from the mean of ΔPWV_ΔPAT Toe-Finger_ in standing for each individual subject. The dashed line indicates the simple linear regression line. The regression equation, the coefficient of determination (R^2^), and P value are presented. PWV: pulse wave velocity; PAT: pulse arrival time; BMI: body mass index; HR: heart rate; MAP: mean arterial pressure.
(TIF)

S4 Fig The comparison between the subjects with high ΔET and those with low ΔET. The subjects were divided into two groups according to their percentile rank of the ΔET_Toe-Finger_: group 1 had lower ΔET_Toe-Finger_ and group 2 had higher ΔET_Toe-Finger_ (A). Each graph showing the distribution of age (B), BMI (C), HR in supine (D), MAP in supine (E), PWV_ΔPAT Toe-Finger_ in supine (F), and ΔPWV_Standing-Supine_ (G) in group 1 and group 2. Each graph represents the individual data points and superimposes a horizontal line at the arithmetic mean, and error bars showing plus and minus one SD. ET: ejection time; BMI: body mass index; HR: heart rate; MAP: mean arterial pressure; PWV: pulse wave velocity; SD: standard deviation.
(TIF)
