# Supplementary figures and images for: Difference between ejection times measured at two different peripheral locations as a novel marker of vascular stiffness
Source: PLoS One. 2017 Nov 29;12(11):e0187781. doi: 10.1371/journal.pone.0187781 (PMC5706705; doi:10.1371/journal.pone.0187781)

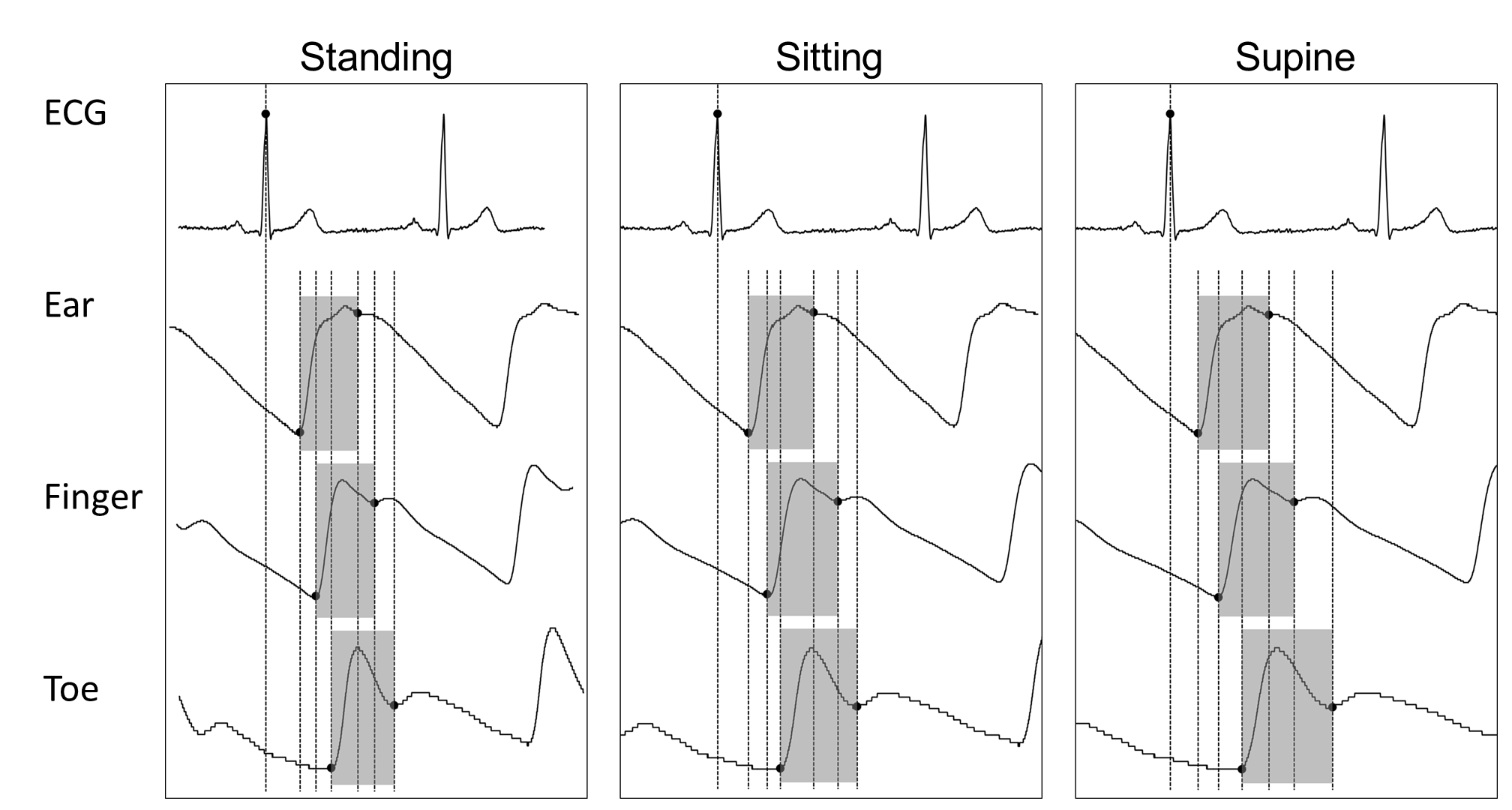

Supplement: S1 Fig — The dots on the electrocardiogram waveforms indicate the peak of R wave, the dots on the plethysmograph waveforms indicate the beginning of the upstroke and dicrotic notch. ET defined between the start of upstroke and dicrotic notch on plethysmograph waveform is shaded gray. The waveform is representative from each location but the duration of ET and dots represent average time calculated from all subjects. ET: ejection time. (TIF) [file pone.0187781.s001.tif]

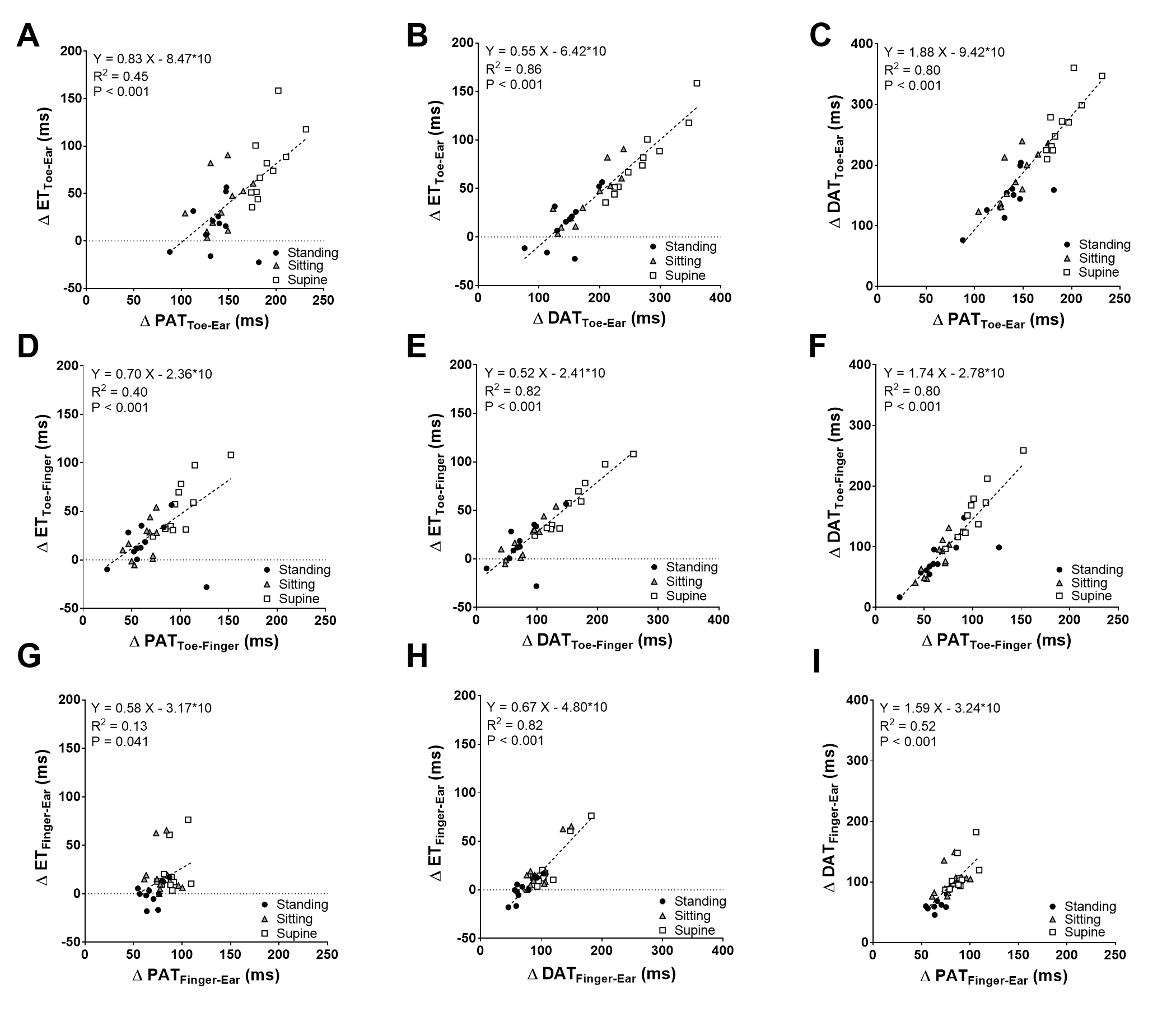

Supplement: S2 Fig — Each individual subject has three dots on the each graph which indicate mean values derived from the standing (black circles), sitting (gray triangles) and supine (white squares) positions. The dashed line indicates the linear regression line. The regression equation, the coefficient of determination (R2), and P value are presented. ET: ejection time; PAT: pulse arrival time; DAT: dicrotic notch arrival time. (TIF) [file pone.0187781.s002.tif]

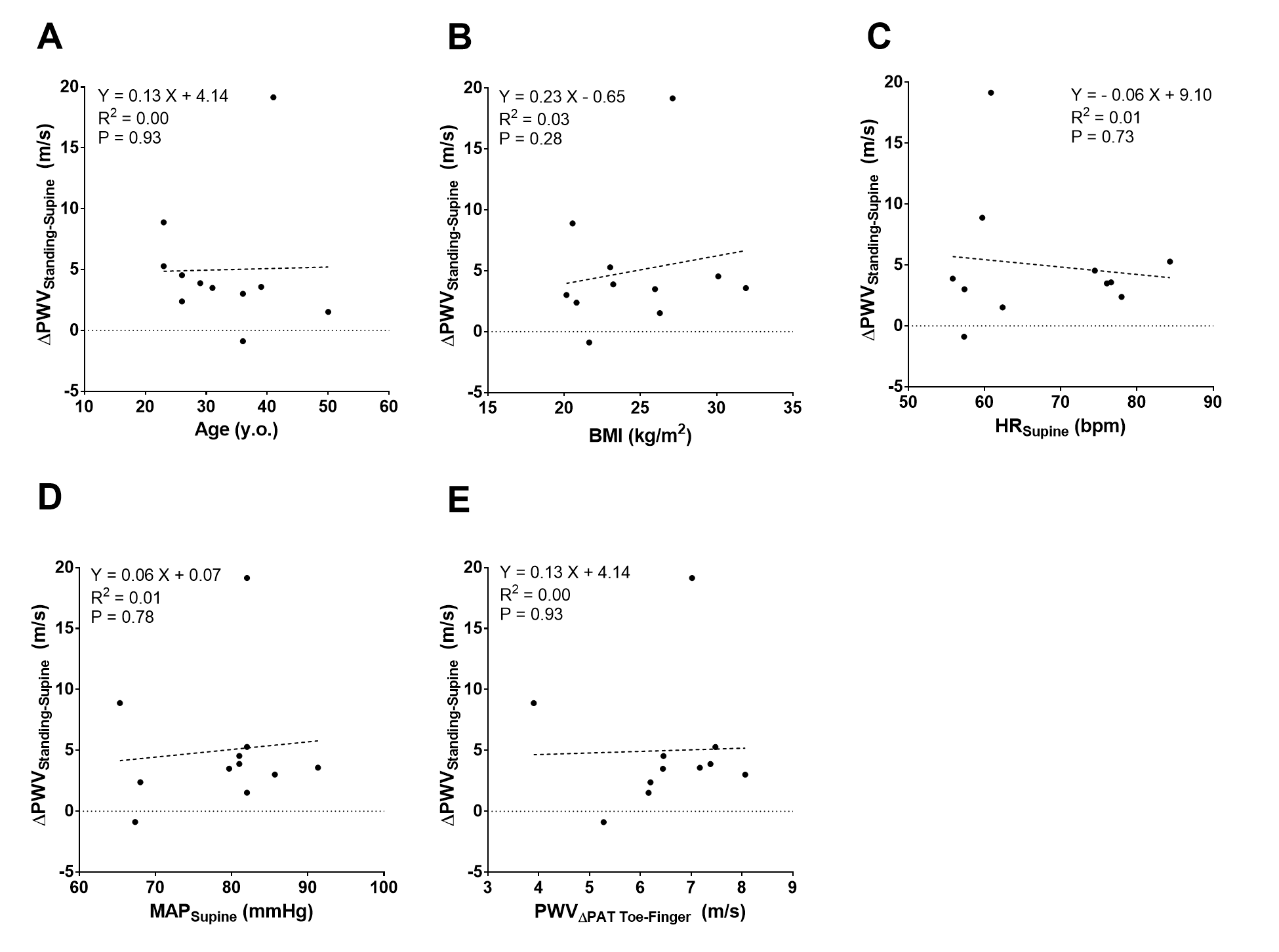

Supplement: S3 Fig — (A) ΔPWVStanding-Supine as a function of age. (B) ΔPWVStanding-Supine as a function of BMI. (C) ΔPWVStanding-Supine as a function of HR measured in supine position. (D) ΔPWVStanding-Supine as a function of MAP measured in supine position. (E) ΔPWVStanding-Supine as a function of PWVΔPAT Toe-Finger in supine position. ΔPWVStanding-Supine was calculated by subtracting the mean of PWVΔPAT Toe-Finger in supine from the mean of ΔPWVΔPAT Toe-Finger in standing for each individual subject. The dashed line indicates the simple linear regression line. The regression equation, the coefficient of determination (R2), and P value are presented. PWV: pulse wave velocity; PAT: pulse arrival time; BMI: body mass index; HR: heart rate; MAP: mean arterial pressure. (TIF) [file pone.0187781.s003.tif]

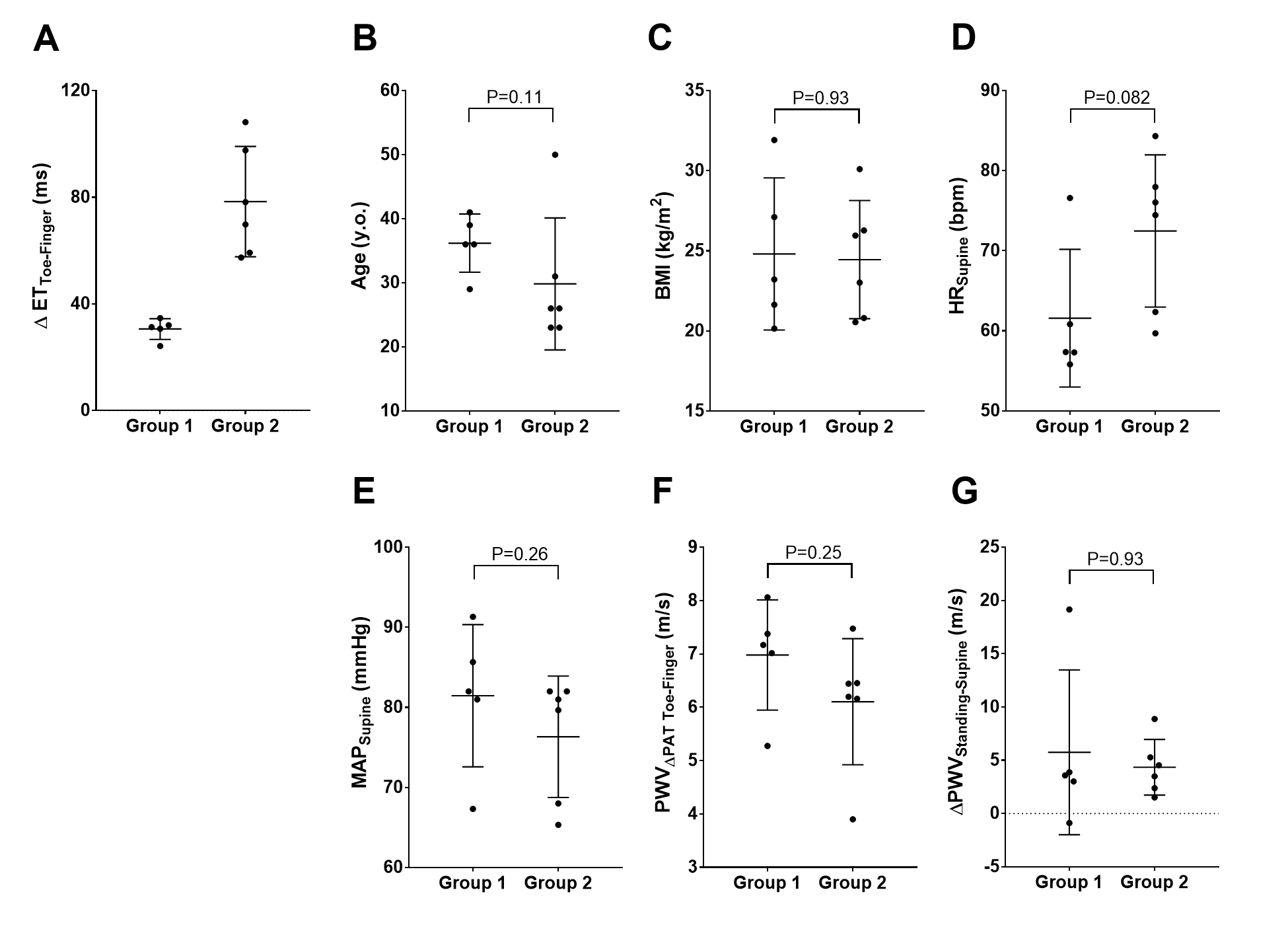

Supplement: S4 Fig — The subjects were divided into two groups according to their percentile rank of the ΔETToe-Finger: group 1 had lower ΔETToe-Finger and group 2 had higher ΔETToe-Finger (A). Each graph showing the distribution of age (B), BMI (C), HR in supine (D), MAP in supine (E), PWVΔPAT Toe-Finger in supine (F), and ΔPWVStanding-Supine (G) in group 1 and group 2. Each graph represents the individual data points and superimposes a horizontal line at the arithmetic mean, and error bars showing plus and minus one SD. ET: ejection time; BMI: body mass index; HR: heart rate; MAP: mean arterial pressure; PWV: pulse wave velocity; SD: standard deviation. (TIF) [file pone.0187781.s004.tif]
